# Food Avoidance and Aversive Goal Value Computation in Anorexia Nervosa

**DOI:** 10.3390/nu16183115

**Published:** 2024-09-15

**Authors:** Siri Weider, Megan E. Shott, Tyler Nguyen, Skylar Swindle, Tamara Pryor, Lot C. Sternheim, Guido K. W. Frank

**Affiliations:** 1 Department of Psychology, Norwegian University of Science and Technology (NTNU), 7491 Trondheim, Norway; 2 Eating Disorder Unit, Department of Psychiatry, Levanger Hospital, Nord-Trøndelag Hospital Trust, 7600 Levanger, Norway; 3 Department of Psychiatry, University of California, San Diego, CA 92093, USA; mshott@health.ucsd.edu (M.E.S.); tmn011@health.ucsd.edu (T.N.); sswindle@health.ucsd.edu (S.S.); 4 ED Care, Denver, CO 80246, USA; tpryor@eatingdisorder.care; 5 Department of Clinical Psychology, Utrecht University, 3584 CS Utrecht, The Netherlands; l.c.sternheim@uu.nl; 6 Rady Children’s Hospital, San Diego, CA 92123, USA

**Keywords:** eating disorders, anorexia nervosa, fMRI, food avoidance, nucleus accumbens

## Abstract

Anorexia nervosa (AN) is associated with food restriction and significantly low body weight, but the neurobiology of food avoidance in AN is unknown. Animal research suggests that food avoidance can be triggered by conditioned fear that engages the anterior cingulate and nucleus accumbens. We hypothesized that the neural activation during food avoidance in AN could be modeled based on aversive goal value processing. Nineteen females with AN and thirty healthy controls matched for age underwent functional magnetic resonance brain imaging while conducting a food avoidance task. During active control free-bid and computer-generated forced-bid trials, participants bid money to avoid eating food items. Brain activation was parametrically modulated with the trial-by-trial placed bids. During free-bid trials, the AN group engaged the caudate nucleus, nucleus accumbens, ventral anterior cingulate, and inferior and medial orbitofrontal cortex more than the control group. High- versus low-bid trials in the AN group were associated with higher caudate nucleus response. Emotion dysregulation and intolerance of uncertainty scores were inversely associated with nucleus accumbens free-bid trial brain response in AN. This study supports the idea that food avoidance behavior in AN involves aversive goal value computation in the nucleus accumbens, caudate nucleus, anterior cingulate, and orbitofrontal cortex.

## 1. Introduction

Anorexia nervosa (AN) is a severe psychiatric illness associated with a restriction of energy intake resulting in low body weight, an intense fear of gaining weight despite being underweight, and a disturbance in body perception [1]. The pathophysiology underlying specific behaviors in AN remains unclear, especially since no data-supported neurobiological models exist for food avoidance in the disorder.

### 1.1. Food Avoidance in AN

Patients with AN show an aversion towards eating food and high-caloric food in particular [2]. Food avoidance has been found to be associated with the severity of the illness, including low BMI and bodily perceptual distortions [2]. Previous research found that individuals with AN showed less “liking” (experiencing pleasure from something) and “wanting” (i.e., craving) in response to conditioned cues that predicted high-caloric foods [3]. Several studies suggested that fear conditioning, a form of aversive learning, is associated with food avoidance [4,5]. Almost 90% of patients with AN reported having food-related aversive learning experiences, 24% reported food-related subjective traumatic experiences [6], and pre-meal anxiety in AN was associated with lower caloric intake [5]. Studies from our group implicated the amygdala in AN when expecting a high caloric stimulus and supported our model of anxiety-related conditioning leading to persistent food avoidance [7,8]. However, how neurobiology is mechanistically involved when individuals with AN try to avoid food remains unclear [9].

### 1.2. Food Avoidance as a Method for Emotion Regulation

AN is associated with elevated trait anxiety and intolerance of uncertainty [10,11,12,13,14], and food avoidance and self-starvation in AN have been suggested to be “a dysfunctional behavior to regulate aversive emotions” [15]. Intolerance of uncertainty, a trait related to anxiety and emotion control [16], is defined as “a dispositional characteristic that results from a set of negative beliefs about uncertainty and its implications and involves the tendency to react negatively on an emotional, cognitive, and behavioral level to uncertain situations and events“ [17]. Anxious traits and deficits in regulating emotions are considered important for the etiology of AN, and studies have suggested that food avoidance reduces the experience of those emotions [18,19,20,21,22].

### 1.3. Brain Activation and Food Avoidance

Preclinical studies in animals from Kent Berridge’s group showed that conditioned environmental ambiance (feeling safe versus under threat) could produce either appetitive (desire) or avoidance (dread) behaviors toward food via the nucleus accumbens, with input from the orbitofrontal cortex for appetitive stimulation, and the infralimbic prefrontal cortex (human anterior cingulate) for aversive and food suppression behaviors [23,24,25,26]. It has been suggested that individuals with AN who are afraid of weight gain learn to fear (are conditioned negatively to) food items and categorize those into safe and unsafe foods with low- or high-calorie content, respectively [4,27]. It is possible that the conditioned fear of high-calorie foods may then trigger the nucleus accumbens dopaminergic circuitry to drive dread and avoidance of food. In AN, there is evidence that those dopaminergic circuits sensitize and facilitate conditioned fear-driven food avoidance via the striatal–hypothalamic food-control circuitry [28]. We postulate that the aversive conditioning toward food in AN encodes aversive goal values across different brain regions to pursue food avoidance and weight loss [29].

When making choices, individuals assign value to the available options and compare those goal values at the time of their decision-making [30,31]. Thus, goal value computation is a critical step in the decision-making process. Appetitive goal values process value-based choices for desirable stimuli, whereas aversive goal values reflect value computations for stimuli the individual would like to avoid. In a functional imaging study on healthy controls, aversive and appetitive goals were localized to the orbitofrontal and prefrontal cortex during decision-making to avoid food [32]. In that study, participants could use money to place bids against a computer (free bids) to avoid eating food items later in the experiment, or the computer placed the bids for them (forced bids). In that study, baby foods or canned meats were used as stimuli. It was hypothesized that the active avoidance free-bid condition may stimulate both goal value computation (instrumental conditioning) but also others, such as disgust-related brain response, which is a threat-related emotion [33]. However, disgust-related brain response, it was hypothesized, could be stimulated in forced-bid trials (classical conditioning), and contrasting free- versus forced-bid trials could further highlight goal value computation-related brain response [34].

The brain circuitry that underlies food avoidance in AN has yet to be systematically tested. One study investigated the neural mechanisms of food choices in recently admitted patients with AN and controls where they had to rate food items according to health value and tastiness or choose preferable food items compared to an item rated as neutral [35]. Results from that study showed a higher striatal response in AN, and connectivity with frontal cortical activation was associated with choosing lower-calorie foods during a later meal. We hypothesized that in individuals with AN, food-related decision-making, especially avoiding high-calorie foods, would help identify the specific underlying neurobiology. Decision-making paradigms in AN with non-food stimuli indicated largely similar performance compared to controls [36]. That finding was perhaps unsurprising since those individuals generally function well in the environment and, in fact, tend to be high achievers [37]. On the contrary, the aversive goal value of avoiding high-caloric food, which is part of the specific AN psychopathology and thus the goal value of preventing weight gain, could be able to engage illness-relevant brain regions. Previously, passive processing of taste value showed greater activation of the anterior cingulate in AN compared to healthy controls [38]. The goal of this study was to test the mechanism of brain activation in AN at the time of processing aversive goal values to actively avoid having to eat food.

In the present study, we used the described food avoidance task [32] adapted to the standard patient diet [32]. We hypothesized that in AN, food avoidance would engage the anterior cingulate (infralimbic prefrontal cortex equivalent) cortex and the nucleus accumbens as an indication of more robust activation in regions that drive dread and avoidance, consistent with the above-described animal model [24]. Further, we wanted to gather support for the hypothesis that food avoidance and related brain responses are associated with emotion regulation in AN.

## 2. Methods

### 2.1. Participants

We recruited 19 female adolescents and young adults with AN (mean age 18.3 ± SD 5.9 years) and 30 female healthy controls (mean age 21.4 ± SD 6.0 years). The Colorado Multiple Institutional Review Board approved the study (#07-0816) and all participants provided written informed consent. Study participants were compensated for their time spent on questionnaires and brain imaging (USD 125). Data gathered were deidentified and stored securely according to local institutional review board requirements. Participants with AN were recruited from eating disorder partial hospitalization specialty care (EDCare Denver or Children’s Hospital Colorado, Denver Colorado, CO, USA) within the first two weeks of treatment, to mitigate the effects of acute starvation or dehydration [39]. Healthy control participants were recruited through local advertisements such as fliers in the Denver, Colorado community. No specific matching procedures were applied except for specific inclusion and exclusion criteria. Participants with AN had no history of other eating disorders and were right-handed without a history of head trauma, neurological disease, major medical illness, bipolar disorder, psychosis, or current (past 3 months) substance use disorder. Healthy control participants did not have a lifetime psychiatric illness, particularly no eating disorder history, did not take medication or have a major medical illness, did not have a first-degree relative with an eating disorder, and had normal lifetime BMI according to the Centers for Disease Control. Menses were absent in 80% of individuals with AN, and controls were studied during the first ten days of the menstrual cycle to reduce hormonal effects.

### 2.2. Behavioral Assessments

A doctoral-level interviewer established participants’ diagnoses using the structured clinical interview for DSM-5 diagnoses (SCID-5) [40]. In addition, the participants completed the following self-report questionnaire package: the Beck Depression Inventory-II (BDI-II) [41] was used as a self-report measure of current depressive symptoms. This 21-item questionnaire is scored on a 4-point Likert scale and provides a summary score of 0 to 63, with higher scores indicating more depressive symptoms. The questionnaire has been found to have good psychometric properties [42]. The Eating Disorder Inventory-3 (EDI-3) [43] was used to investigate eating disorder-related thoughts and behaviors. The EDI-3 is a 91-item questionnaire scored on a 6-point Likert scale, providing 12 subscales and 6 composite scores. For the current purpose, the subscales of body dissatisfaction, bulimia tendencies, drive for thinness, and emotion dysregulation were used. The EDI-3 has been found to have acceptable psychometrics across different countries [43,44,45,46,47]. The State–Trait Anxiety Inventory (STAI) [48] was used to measure participants’ levels of state and trait anxiety, respectively. The STAI includes 20 items and is scored on a 4-point Likert scale. The Intolerance of Uncertainty Scale (IUS) [49] measured the participants’ reactions, attitudes, and beliefs about future events, ambiguity, and uncertainty. The IUS includes 27 items and is scored on a 5-point Likert scale. The questionnaire has shown good psychometric properties [50].

### 2.3. Brain Imaging

#### 2.3.1. fMRI Image Acquisition

Participants were assessed for height and weight (Health O Meter 500KL Digital Physician Scale; Pelstar, LLC, Countryside, IL, USA) and were served a standard breakfast based on the typical AN treatment program breakfast in our setting, which includes all food groups. The food on the participants’ plates was weighed before and after breakfast, and the percentage of individual food items eaten was used to calculate the calories consumed. The instruction was to eat until they felt full. Furthermore, participants were instructed to have their usual morning coffee or tea. Functional magnetic resonance brain imaging (fMRI) was performed between 0800 and 0900 h (GE Signa or Siemens Skyra 3T scanner), a sagittally acquired, spoiled gradient sequence T1 weighted, 172 slices (thickness = 1 mm, TI = 450 ms, TR = 8 ms, TE = 4 ms, flip angle = 12°, FOV = 22cm, scan matrix = 64 × 64, and T2*-weighted echo-planar scans for blood-oxygen-level-dependent (BOLD) functional (fMRI) activity (3.4 × 3.4 × 2.6 mm voxels, TR = 2250 ms, TE = 30 ms, flip angle = 70°, 34 axial slices, thickness = 2.6 mm, gap = 1.4 mm).

#### 2.3.2. Food Avoidance Task

The task, previously described by Plassmann et al., was adapted in our lab and presented using E-Prime (Psychology Software Tools, Pittsburgh, PA, USA). During fMRI, 2-dimensional (2D) pictures of 35 typical snack foods were presented individually under two conditions (free or forced bids). The participant’s task was to avoid food items based on their aversion to eating that snack food. These foods were either low, medium, or high in caloric content. Examples of included foods were apple sauce, dried fruit, yogurt (low caloric content), mixed nuts, hard-boiled eggs, cookies (medium caloric content), bagels with butter, bread with peanut butter, and protein bars (high caloric content). Participants were told to play a game against the computer where they would be asked to place monetary bids to avoid having to eat one of the snack foods for which participants had lost the bid later in the study. Those were the free-bid trials in the experiment to reflect a situation where the individual has a choice. The randomly chosen snack from the lost trials was added to a person with AN’s meal plan and had to be eaten as their evening snack that day. That time was selected since meal plan changes had to be coordinated with the treatment team. Healthy controls had to eat the snack immediately after the scan to ensure that the snack was in fact eaten. If participants bid correctly, they could avoid eating unwanted food items. In the fMRI scanner, they were told they would have a total of USD 90 ‘play’ money at their disposal, with the directions that they could bid USD 1, USD 2, USD 3, or USD 4, contingent on how much they wanted to avoid eating that food (higher USD amount bid reflecting higher aversive goal value). The total amount of money was limited, so a person could only bid USD 4 for some but not all food items and had to choose based on aversive goal value. In addition to the free-bid trials (control over monetary bid and thus actively driving food avoidance), there were also forced-bid trials in which participants had to bid a computer-determined amount (no control over the bid) to avoid the food presented. Thus, in the forced-bid trials, study participants were not free to choose and had to accept the outcome. In high level of care (HLOC) treatments, individuals with AN are frequently forcibly fed, and are not able to choose what they do eat and what they do not. The forced-bid trial response, therefore, was considered to reflect emotions such as disgust, but not the decision-making process leading to active avoidance [32]. The free-bid response, on the other hand, was hypothesized to encompass brain activation that reflects both aversive goal value computation for active avoidance but also other factors, such as disgust toward a particular food. It was further hypothesized that subtracting the forced bid (passive emotional response) from the free bid (aversive goal value computation for active avoidance plus passive emotional response) would more specifically identify brain response associated with aversive goal value computation leading to active food avoidance.

#### 2.3.3. fMRI Analysis

Image preprocessing and analysis were performed using SPM12 (http://www.fil.ion.ucl.ac.uk, Accessed on 2 November 2023), including image realignment, normalization (Montreal Neurological Institute template), smoothing (6 mm FWHM), slice time correction, and modeling with a hemodynamic response convolved function (general linear model, temporal and dispersion derivatives added). Each image sequence was inspected, and images with movement > one voxel size were removed. A128-s high-pass filter and SPM FAST were applied to account for low-frequency fluctuation autocorrelations [51]. Motion parameters and missed trials were included as regressors of no interest.

Plassmann’s study initially investigated whole brain activation, but due to a lack of significant results after multiple comparison corrections, they used an ROI-based approach. We, therefore, extracted mean beta values from predefined regions of interest (Automated Anatomical Labeling Atlas [52]) using MarsBar [53] as the primary analysis method, which is a best-practice approach [54]. Regional data were extracted for the ventral anterior cingulate cortex, the orbitofrontal cortex, the head of the caudate nucleus, and the nucleus accumbens. Those regions were selected based on brain regions identified in the described animal studies and Plassmann’s study [23,24,25,26,32].

A general linear model, including the regressors ‘free-bid at response’ and ‘forced-bid at response’, was applied to the data. The regressors for the bid responses were modeled as events with a duration equal to the participant’s response time (measured from the time of appearance of the food item on the bid screen). The bid responses were used as parametric modulators of the brain response. The following contrasts were calculated: (1) correlation with aversive goal values in free trials, (2) correlation with aversive goal values in forced-bid trials, and (3) correlation with aversive goal values in free versus forced trials. The contrast free-minus forced-bid trials was computed to remove activation that was less due to aversive goal value computation (instrumental conditioning) but other factors such as disgust (classical conditioning), as discussed previously.

We further conducted an exploratory whole-brain analysis of the HC group to validate results in comparison to the original study design. In this study, we modified the original study design previously described by Plassmann et al. [32] and replaced the food items used in the original study (such as baby foods and canned meats) with snack food items that individuals typically encounter in everyday life, including in their treatment program. To validate and compare our modified study design with the previous study, we conducted an exploratory free-bid whole-brain analysis in the HC group. Whole brain images were normalized using the statistical parametric mapping software (SPM12) to the MNI template. Parametric images were created, and free-bid monetary values were correlated with associated brain response during bidding and thus aversive goal value processing to avoid having to eat food. One-sample t-tests were conducted (cluster threshold 10 voxels, *p* = 0.005) to test significant positive or negative correlations between brain activation and monetary bids.

### 2.4. Statistical Analysis

All analyses were conducted using SPSS 29 software (IBM, Armonk, NY, USA). Data were tested for normality with Shapiro–Wilk tests. Behavioral and brain imaging data were non-normally distributed and ranked data, or non-parametric tests were used in group contrast and correlation analyses. Behavioral data were assessed using a student’s *t*-test. Sensitivity analyses tested for potential confounding variables such as comorbidity or medication use and were included in the statistical model if indicated. Age and scanner were included as covariates regardless. Brain imaging group contrasts were calculated using MANCOVA (ranked data), and the Quade Non-Parametric ANCOVA using unranked data was used to confirm the regional MANCOVA results. Correlation analyses between brain region activation and symptoms were conducted using Pearson correlation analysis (ranked data) and false discovery rate (FDR) corrected. Tests were considered statistically significant at a *p*-value < 0.05. Partial eta squared (η2) values were calculated for group contrasts as a measure of effect size (0.01 small effect size, 0.06 medium effect size, 0.14 or greater large effect size).

## 3. Results

### 3.1. Demographics and Behavioral Data

Among the AN group, 15 participants had restrictive AN, 3 had AN with purging, and 1 participant had AN with binge eating and purging behaviors. The AN group was slightly younger than the HC group without statistical significance (Table 1). The AN group scored higher on the BDI-II and EDI-3 scales, intolerance of uncertainty (IUS), and state and trait anxiety (STAI). In the AN group, 18 identified as White and one as American Indian/Alaska Native; in the HC group, 24 identified as White, 3 as Asian, and 2 as More than One Race. One person in the AN and three in the HC group identified as LatinX.

Participant distribution across the two scanners was similar across groups, with 23/30 of the HC and 15/19 of the AN group scanned on the Siemens Skyra scanner (χ^2^ = 0.035, *p* = 0.852). Scanner type was not significantly associated with brain activation within groups and across trial types (*p* > 0.05), indicating no scanner effect. Nevertheless, age and scanner were included as covariates in the group comparison for regional activation during food avoidance and goal value processing to account for effects not detected due to small groups and lack of power. Comorbidity or medication use was not significantly associated with brain response during goal value computation and, therefore, was not included in the model.

### 3.2. Goal Value Computation across Groups

**Figure 1:**
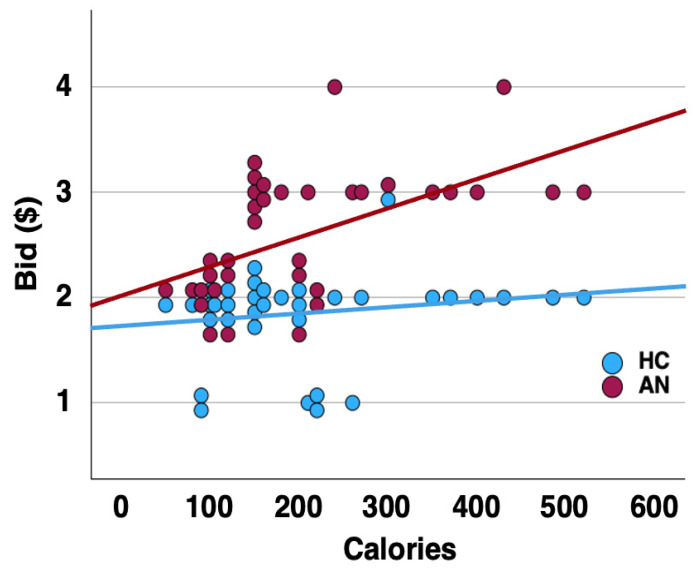
Correlation slope for the calorie content of the thirty-five food items shown and mean monetary bids placed across groups.

The mean value for money spent per bid by the participants in the AN group (USD 2.60 ± 0.60) was significantly higher than in the controls (USD 1.86 ± 0.43; t = −5.98, *p* < 0.001). In the AN group, there was a significant correlation between food item calories and bids placed (rho = 0.642, *p* < 0.001) but not in the healthy control group (rho = 0.078, *p* = 0.655). This difference was significant between groups (Fisher Z = −2.17, *p* = 0.03) (Figure 1).

#### Region of Interest Analysis

Brain imaging group comparison was conducted in two ways to account for data distribution and inclusion of covariates. First, a MANCOVA (covariates age and scanner) was conducted using ranked data. While using ranked data in interaction models (factorial ANOVA) can lead to distortions, here, we used only one independent variable, which is acceptable [55]. However, to validate the results, we conducted a Quade Non-Parametric ANCOVA on significant MANCOVA results.

Free-bid trials (Table 2): The MANCOVA results showed a significant overall group effect with Wilk’s lambda = 0.491, F = 2.942, *p* = 0.007, a very large effect size, partial η2 = 0.509, and moderate power of 0.596. The AN group showed greater engagement during aversive goal value processing during free-bid trials for the right ventral anterior cingulate, left inferior and right medial orbitofrontal cortex, bilateral caudate head, and left nucleus accumbens. The Quade Non-Parametric ANCOVA (covariates age and scanner) confirmed significant results for the right anterior ventral cingulate cortex (F = 4.258, *p* = 0.045), the left inferior orbitofrontal cortex (F = 4.969, *p* = 0.031), the right caudate head (F = 4.255, *p* = 0.045), the left caudate head (F = 11.140, *p* = 0.002), and the left nucleus accumbens (F = 5.472, *p* = 0.024).

Forced-bid trials: There was no overall MANCOVA group effect (Wilks Lambda = 0.739, F = 1.001, *p* = 0.468), but the effect size was large, part η2 = 0.261, and the power of 0.5 was moderate. The left nucleus accumbens contrast showed significantly higher values in the AN group than in the controls, but the Quade Non-Parametric ANCOVA (covariates age and scanner) indicated a non-significant result (F = 4.040, *p* = 0.05).

Free-minus forced-bid trials: There was no overall MANCOVA group effect (Wilks Lambda = 0.0.688, F = 1.284, *p* = 0.468), but the effect size was large, part η2 = 0.272, with a moderate power of 0.6. Participants with AN did not show significantly higher regional brain response than controls across individual brain regions.

A post hoc power analysis for the free-bid trials’ significant regional group differences indicated for the left caudate head a very large effect size of partial η2 = 0.222 and power of 0.95; for the right caudate head, a moderate-to-large effect size of partial η2 = 0.104 and power of 0.7; for the left nucleus accumbens, a moderate-to-large effect size partial η2 = 0.115 and power of 0.7; for the right anterior cingulate, a moderate-to-large effect size of partial η2 = 0.092 and power of 0.6; for the inferior orbitofrontal cortex left, a moderate-to-large effect size of partial η2 = 0.088 and power of 0.6; and for the medial orbitofrontal cortex right, a moderate-to-large effect size of partial η2 = 0.0.083 and power of 0.5.

The exploratory whole-brain analysis in the HC group showed negative correlations between placed free bids and the medial orbitofrontal cortex, insula, and caudate, but results were non-significant on the voxel or cluster level after FDR or FWE multiple comparison correction.

### 3.3. Differential Brain Response to Low and High-Bid Trials

**Figure 2:**
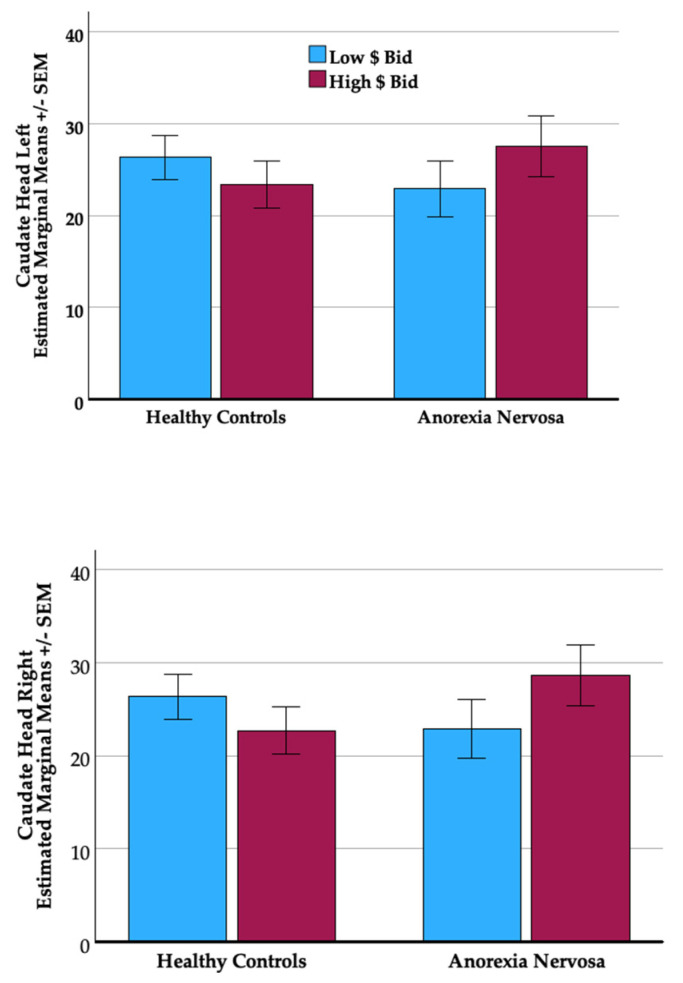
Brain response to low and high bid across the study groups in the caudate nucleus.

To test whether higher food avoidance efforts in AN were associated with higher brain response, we separated bids in the free-bid condition in low (USD 1 and USD 2) and high (USD 3 and USD 4) bid conditions and tested associated brain response. Low and high bid response is displayed for bilateral caudate head in Figure 2 (ranked data). An exploratory two-group by two-condition ANCOVA that included age and scanner as covariates indicated significant group-by-condition results for the left (unranked F = 5.557, *p* = 0.023, η2 = 0.110; ranked F = 5.572, *p* = 0.023, η2 = 0.110) and right (unranked F=5.196, *p* = 0.027, η2 = 0.104; ranked F = 6.865, *p* = 0.012, η2 = 0.132) caudate head. Patterns of results were similar for the anterior cingulate, nucleus accumbens, and orbitofrontal cortex across groups but they were non-significant (see Supplementary Materials Figure S1).

### 3.4. Demographics and Behavior—Brain Response Correlations

In AN, BMI was negatively correlated with free-bid aversive goal value processing in the left nucleus accumbens (rho = −0.475, *p* = 0.040). Age correlated negatively with the free-bid right middle orbitofrontal cortex (rho = −0.502, *p* = 0.029) and the bilateral middle orbitofrontal cortex response (R:rho = −0.537, *p* = 0.018; L:rho = −0.460, *p* = 0.047).

In HC, BMI correlated positively with bilateral medial orbitofrontal cortex free-bid response (R:r = 0.373, *p* = 0.042; L:r = 0.397, *p* = 0.030).

In AN, EDI-3 emotion dysregulation in free-bid trials correlated negatively with left nucleus accumbens aversive goal value processing, and intolerance of uncertainty correlated negatively with left ventral anterior cingulate cortex, caudate, and nucleus accumbens free-bid trial response (Table 3). There were no significant behavior correlations with forced-bid trials (Supplemental Table S2). For the contrast free-minus forced-bid trials, emotion dysregulation was negatively correlated with the left caudate nucleus (r = −0.645, q = 0.047) and nucleus accumbens response (r = −0.788, q = 0.003; Supplemental Table S4).

In HC, there were positive associations between EDI-3 emotion dysregulation and free-bid aversive goal value processing in the right ventral anterior cingulum (r = 0.425, q = 0.048), caudate head (r = 0.547, q = 0.023), and nucleus accumbens (R:r = 0.524, q = 0.025; L:r = 0.460, q = 0.035). There were no significant behavior correlations with forced-bid trials or free- minus forced-bid trials (Supplemental Tables S1, S3, and S5).

## 4. Discussion

The present study indicates that, in AN, active food avoidance and thus aversive goal value processing engages the caudate nucleus, nucleus accumbens, anterior cingulate, and orbitofrontal cortex brain response more strongly than in healthy controls. Stronger neural goal value processing in those regions was associated with lower intolerance of uncertainty and emotion dysregulation in AN. In contrast, brain response was associated with higher emotion dysregulation scores in the HC group. The results are consistent with previous animal research that has implicated the anterior cingulate and nucleus accumbens in conditioned aversive or dread response toward food. The data further support that food avoidance may have a role in regulating negative emotions in AN.

Food avoidance, self-starvation, and low body weight are hallmark signs of AN [1]. Previous animal research has suggested that the infralimbic prefrontal cortex, which is homologous to the human anterior cingulate, and the nucleus accumbens have a central role in dread and avoidance response to food when in a negatively conditioned environment [56]. A human brain imaging study in healthy controls emphasized goal value encoding in the orbitofrontal and prefrontal cortex when trying to avoid eating certain foods [32]. A previous study from our group found altered anterior cingulate response in AN compared to healthy controls during passive food-related value processing [38]. The current study tested brain function while study participants actively tried to avoid having to eat food later in the experiment. The ROI-based group contrasts in our sample for free-bid response indicated an overall elevated response in AN. Regional goal value processing in AN was more strongly associated with nucleus accumbens, caudate, anterior cingulate, and orbitofrontal cortex response than in controls. Forced- and free-minus forced-bid conditions did not show overall group differences or regional differences after confirmatory tests.

During free-bid trials, participants had control over the amount of their bids, whereas they were required to bid a computer-generated amount in the forced-bid trials. Individuals with AN are conditioned to respond with fear to high-caloric food stimuli and often separate foods into safe and unsafe food items [27]. We postulate that activation during free-bid trials in AN is driven by negative conditioning and “feeling unsafe” around high-calorie foods. Kent Berridge and colleagues have shown that feeling unsafe triggers dread and avoidance toward food via caudal nucleus accumbens dopaminergic D1 and D2 receptors with input from the anterior cingulate, which is the human equivalent to the rodent infralimbic prefrontal cortex [23,24,25,26]. We believe that conditioned fear and associated aversive goal values in AN trigger the avoidance response to food stimuli via the anterior cingulate, ventral striatum, and orbitofrontal cortex [4,57], which is consistent with the animal model [58].

The significant overall and regional group contrasts in our sample were found for the free-bid trials only. The lack of group differences in the forced-bid trials supports that, in particular, during trials with active control brain response in AN engaged more than in controls, and the other factors hypothesized to contribute to brain response, such as disgust, may play less of a role. Clinical observation suggests that patients with AN can weight-restore in a highly structured environment and thus with enforced food intake but may quickly return to self-starvation when they are on their own, and fears of weight gain drive their behaviors [59]. This contingency underlines the importance of a gradual transfer of control of eating and weight control from the treatment facility to the individual and the significance of long-term treatment of these patients to modify goal values, such as developing life goals to counter exaggerated fears of eating and weight gain [60].

In the control group, higher emotion dysregulation was associated with higher brain response during free-bid goal value processing in the anterior cingulate, caudate, and nucleus accumbens. It is possible that healthy controls with higher emotion dysregulation are more responsive in those brain regions when processing aversive goals and thus respond strongly. In the AN group, the pattern was the opposite. Here, EDI-3 emotion dysregulation correlated negatively with the left nucleus accumbens free-bid response, and intolerance of uncertainty was negatively correlated with the free-bid response across the right inferior orbitofrontal gyrus, left caudate, and nucleus accumbens. Previously, it was hypothesized that self-starvation could be a means to regulate negative emotions in AN [15]. Intolerance of uncertainty is a behavioral trait associated with an elevated tendency to worry and a decreased capacity to regulate negative emotions. The association of lower intolerance of uncertainty and emotion dysregulation with higher brain response in AN suggests that higher aversive goal processing and, thus, efforts to avoid food intake may help reduce negative affect and emotion dysregulation, supporting the model described by Brockmeyer et al. [15].

**Figure 3:**
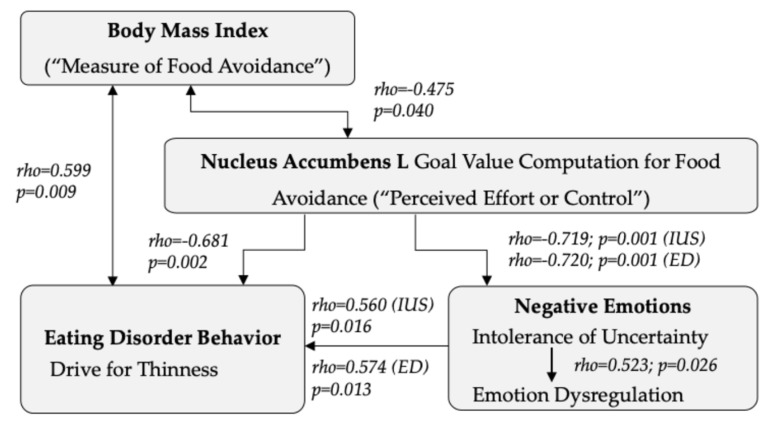
Higher left (L) nucleus accumbens free-bid goal value computation to avoid high caloric foods was associated with lower BMI and drive for thinness; nucleus accumbens aversive goal value computation was also associated with lower scores on the Intolerance of Uncertainty Scale (IUS), and Eating Disorder Inventory Emotion Dysregulation Subscale (EDI-ED).

The negative relationships between nucleus accumbens response in AN during food avoidance with BMI, intolerance of uncertainty, and emotion dysregulation, but a positive relationship with the EDI-3 drive for thinness, lend themselves to the hypothesis that higher aversive goal value computation during food avoidance in the nucleus accumbens is reflected in lower BMI as a measure of being able to avoid food successfully. At the same time, stronger brain responses and more successful food avoidance may help individuals feel in control and able to control intolerance of uncertainty and emotion dysregulation (Figure 3).

### Strengths and Limitations

This study provides the first evidence of specific brain regions directly involved in decision-making related to food avoidance in AN. The study participants were rigorously assessed, and the individual’s behavior response was integrated into the brain imaging data analysis. Results were tested for multiple comparisons. The data lend themselves to connecting AN core signs and symptoms of low body weight and drive for thinness with nucleus accumbens brain response and emotion regulation. Several limitations must be considered. While effect sizes were moderate to large, the group sizes were modest, and larger groups may have shown different results. Group differences showed in particular large differences and a very well-powered sample for the free-bid left caudate head response; however, while for other regions results indicated moderate-to-large effect sizes, power was below the desired level and the study requires replication in a larger sample. To compare the results of this study with Plassmann’s study, we also conducted an exploratory whole-brain analysis. We found similar patterns of negative correlations between placed free bids and the medial orbitofrontal cortex, insula, and caudate. Those results indicate that the results in the HC group are consistent with the original study; however, results were not significant after multiple comparison corrections, a frequent shortcoming of whole-brain analysis. Larger samples would be needed for significant whole-brain contrasts, which is frequently prohibitive in difficult-to-recruit samples. Therefore, we selected to present ROI-based data similar to Plassmann et al.’s study [32]. ROI-based analyses are considered a best-practice approach and are particularly important when comparing healthy with psychiatric groups where effect sizes tend to be small [39,54]. The study focused on aversive goal values and food avoidance in AN, and how this relates to reward processing or appetitive value processing requires further study [61]. To relate to real-life circumstances and food that is avoided in AN, we did not use food that was aversive to controls. Thus, fewer aversive goal values might have been assigned to the food in controls, limiting the comparability between participants with AN and controls. Yet, the study’s focus was on AN psychopathology and we believe that the study design using typical food items was appropriate. The time for snacking on one of the foods for which the individual had lost the bid was after the scan in the late morning for healthy controls, but it was in the evening for the individuals in the AN group to ensure supervision of controls and coordination with meal plan procedures in the AN group. We have no evidence to suggest that the difference in time of snack consumption between groups affected the study outcome, but effects can also not be excluded. Using 2D images of food items might also have affected the results since research has found differences in the processing of 2D and real-world 3D stimuli [62]. The included patients were relatively young, and the results may not transfer to an older population. In addition, while we explored potentially confounding variables that did not show significant effects, this could have been due to the modest sample size. We included age and scanner in the group comparisons regardless. Furthermore, the analysis could not distinguish effects between AN subtypes.

## 5. Conclusions

Food avoidance and related aversive goal value processing of food stimuli in AN can be linked to striatal and cortical brain regions, including the nucleus accumbens and caudate head, the anterior cingulate, and the orbitofrontal cortex. Our results are consistent with animal studies that implicated those regions in dread and avoidance response to food. The results also support earlier reports that food avoidance helps attenuate emotion dysregulation. The nucleus accumbens food avoidance mechanisms, which involve specific dopaminergic receptors, could be targeted with pharmacologic interventions and could become targets to treat self-starvation in AN. Future research should systematically explore whether brain response in regions such as the anterior cingulate and caudate nucleus can be identified as biomarkers for the severity of food avoidance, whether decision-making and emotional responses such as disgust can be further separated by specific brain circuitry, and whether brain response can be used as an objective marker to test the effectiveness of pharmacological or behavioral interventions.

## Figures and Tables

**Table 1:** Demographic and behavioral data in the two study groups. AN, anorexia nervosa; HC, healthy controls. BMI, Body Mass Index; BDI-II, Beck Depression Inventory-II; EDI-3, Eating Disorder Inventory 3; STAI, State–Trait Anxiety Inventory.

	HC (n = 30)		AN (n = 19)		t	*p*
	Mean	SD	Mean	SD		
Age in Years	21.43	5.64	18.25	5.89	1.89	0.065
BMI (kg/m^2^)	20.69	1.77	16.18	1.07	9.99	<0.001
BDI-II Sum	1.35	2.23	2.5	14.81	−4.92	<0.001
EDI-3 Drive for Thinness	1.90	2.38	17.94	8.65	−7.70	<0.001
EDI-3 Bulimia	0.86	1.09	4.28	6.76	−2.13	0.048
EDI-3 Body Dissatisfaction	4.17	4.46	24.24	11.85	−6.71	<0.001
EDI-3 Emotion Dysregulation	1.28	2.07	4.83	4.87	−2.94	0.008
Intolerance of Uncertainty	46.93	16.89	73.21	22.02	−4.71	<0.001
STAI State Anxiety	26.60	6.18	49.16	13.88	−6.68	<0.001
STAI Trait Anxiety	27.53	5.11	51.68	15.05	−6.75	<0.001
Breakfast Calories (kcal)	648.84	124.88	585.7	154.2	1.55	0.129
			N	%		
Major Depressive Disorder			11	57.9		
Anxiety Disorder			12	63.2		
Antidepressant Use			10	52.6		
Antipsychotic Use			2	10.5		

**Table 2:** Goal value computation response by trial condition, brain regions, and across groups. AN, anorexia nervosa; HC, healthy controls; MANCOVA including covariates age and scanner.

	Free-bid trials							Forced-bid trials							Free- minus forced-bid trials						
	HC (n = 30)		AN (n = 19)		F	p	partial η2	HC (n = 30)		AN (n = 19)		F	p	partial η2	HC (n = 30)		AN (n = 19)		F	p	partial η2
	Mean	SE	Mean	SE				Mean	SE	Mean	SE				Mean	SE	Mean	SE			
Ventral anterior cingulate cortex, right	21.4	2.6	30.7	3.3	4.575	**0.038**	0.092	23.3	2.7	27.7	3.5	0.953	0.334	0.021	22.9	2.6	28.3	3.4	1.505	0.226	0.032
Ventral anterior cingulate cortex, left	22.1	2.7	29.6	3.4	2.834	0.099	0.059	23.5	2.7	27.3	3.5	0.708	0.405	0.015	23.5	2.6	27.3	3.4	0.760	0.388	0.017
Inferior orbitofrontal cortex, right	24.3	2.7	26.1	3.5	0.166	0.686	0.004	25.1	2.7	24.9	3.4	0.001	0.971	0.000	24.3	2.7	26.0	3.4	0.141	0.709	0.003
Inferior orbitofrontal cortex, left	21.5	2.6	30.5	3.3	4.336	**0.043**	0.088	25.3	2.7	24.5	3.4	0.040	0.842	0.001	23.1	2.6	28.0	3.3	1.277	0.264	0.028
Medial orbitofrontal cortex, right	21.6	2.6	30.4	3.4	4.078	**0.049**	0.083	22.2	2.7	29.4	3.4	2.640	0.111	0.055	24.2	2.7	26.2	3.5	0.179	0.674	0.004
Medial orbitofrontal cortex, left	22.9	2.7	28.2	3.4	1.399	0.243	0.030	22.5	2.7	29.0	3.4	2.117	0.153	0.045	25.6	2.7	24.1	3.5	0.101	0.752	0.002
Middle orbitofrontal cortex, right	25.5	2.7	24.3	3.4	0.069	0.793	0.002	27.4	2.6	21.2	3.4	1.987	0.166	0.042	23.9	2.6	26.7	3.4	0.416	0.522	0.009
Middle orbitofrontal cortex, left	25.3	2.7	24.5	3.5	0.037	0.849	0.001	26.4	2.7	22.8	3.4	0.651	0.424	0.014	24.7	2.6	25.5	3.4	0.033	0.858	0.001
Caudate head, right	21.1	2.6	31.1	3.3	5.245	**0.027**	0.104	24.1	2.6	26.4	3.3	0.275	0.603	0.006	22.3	2.6	29.2	3.4	2.440	0.125	0.051
Caudate head, left	19.6	2.3	33.6	3.0	12.830	**0.001**	0.222	24.3	2.6	26.1	3.3	0.173	0.679	0.004	22.1	2.5	29.5	3.2	3.047	0.088	0.063
Nucleus accumbens, right	21.6	2.6	30.3	3.4	3.873	0.055	0.079	23.2	2.6	27.9	3.3	1.215	0.276	0.026	24.2	2.7	26.3	3.5	0.211	0.648	0.005
Nucleus accumbens, left	21.0	2.6	31.4	3.3	5.822	**0.020**	0.115	21.4	2.5	30.7	3.2	4.881	**0.032**	0.098	24.6	2.7	25.7	3.4	0.061	0.807	0.001

Note: significant values are in bold. The Quade Non-parametric ANCOVA (covariates age and scanner) confirmed significant results for the free-bid trials in the right anterior ventral cingulum (F = 4.258, *p* = 0.045), left inferior orbitofrontal cortex (F = 4.969, *p* = 0.031), right caudate head (F = 4.255, *p* = 0.045), left caudate head (F = 11.140, *p* = 0.002), and left nucleus accumbens (F = 5.472, *p* = 0.024). Quade Non-parametric ANCOVA was not significant for forced-bid trial left nucleus accumbens result.

**Table 3:** Correlation between brain activation and emotion regulation measures in the anorexia nervosa (AN) group in free-bid trials.

	EDI-3 ED		IUS	
	r	q	r	q
Ventral anterior cingulate, right	−0.253	0.393	−0.368	0.218
Ventral anterior cingulate, left	−0.414	0.178	**−0.570**	**0.040**
Inferior orbitofrontal gyrus, right	−0.379	0.219	−0.308	0.262
Inferior orbitofrontal gyrus, left	−0.091	0.728	−0.305	0.262
Medial orbitofrontal gyrus, right	−0.326	0.280	−0.351	0.230
Medial orbitofrontal gyrus, left	−0.257	0.393	−0.210	0.404
Middle orbitofrontal gyrus, right	−0.231	0.395	−0.211	0.404
Middle orbitofrontal gyrus, left	−0.542	0.099	−0.325	0.261
Caudate head, right	−0.365	0.225	−0.414	0.158
Caudate head, left	−0.603	0.063	**−0.616**	**0.034**
Nucleus accumbens, right	−0.467	0.133	−0.279	0.294
Nucleus accumbens, left	**−0.697**	**0.017**	**−0.556**	**0.042**

Note: bolded values are significant after multiple comparison adjustments (FDR-corrected, q-values). Abbreviations: EDI-3, Eating Disorder Inventory-3; ED, emotion dysregulation; IUS, intolerance of uncertainty.

## Data Availability

The authors will make the raw data supporting this article’s conclusions available upon request.

## Supplementary Materials

The following supporting information can be downloaded at: https://www.mdpi.com/article/10.3390/nu16183115/s1, Figure S1. Brain response to low and high bid across the study groups in anterior cingulate and nucleus accumbens. Table S1: correlation between brain activation and behavior in the healthy control (HC) group in free-bid trials with FDR-correction (q); Table S2: correlation between brain activation and behavior in the group with anorexia nervosa (AN) in forced-bid trials with FDR-correction (q); Table S3: correlation between brain activation and behavior in the healthy control (HC) group in forced-bid trials with FDR correction (q); Table S4: correlation between brain activation and behavior in free-minus forced-bid trials in the AN group with FDR correction (q); Table S5: correlation between brain activation and behavior in free-minus forced-bid trials in the healthy control (HC) group with FDR correction (q).
