# Supplementary material for: Food Avoidance and Aversive Goal Value Computation in Anorexia Nervosa
Source: Nutrients. 2024 Sep 15;16(18):3115. doi: 10.3390/nu16183115 (PMC11434691; doi:10.3390/nu16183115)

Supplemental Material

Supplemental Table S1. Correlation between brain activation and behavior in the healthy control (HC) group in free-bid trials with FDR-correction (q).

		EDI-3 ED	IUS
**Ventral anterior cingulum, right**	r	**0.425**	0.047
	q	**0.048**	0.923
**Ventral anterior cingulum, left**	r	0.311	0.053
	q	0.148	0.923
**Inferior orbito-frontal gyrus, right**	r	0.415	0.143
	q	0.051	0.923
**Inferior orbito-frontal gyrus, left**	r	0.264	0.158
	q	0.209	0.923
**Medial orbitofrontal gyrus, right**	r	0.140	-0.155
	q	0.478	0.923
**Medial orbitofrontal gyrus, left**	r	0.348	0.031
	q	0.104	0.924
**Middle orbito-frontal gyrus, right**	r	0.238	0.084
	q	0.238	0.923
**Middle orbito-frontal gyrus, left**	r	0.237	0.078
	q	0.238	0.923
**Caudate head, right**	r	**0.547**	-0.044
	q	**0.023**	0.923
**Caudate head, left**	r	0.284	-0.051
	q	0.184	0.923
**Nucleus accumbens, right**	r	**0.524**	0.011
	q	**0.025**	0.955
**Nucleus accumbens, left**	r	**0.460**	0.077
	q	**0.035**	0.923

Abbreviations. EDI-3, Eating Disorder Inventory-3; ED, Emotion Dysregulation; IUS, Intolerance of Uncertainty.

Supplemental Table S2. Correlation between brain activation and behavior in the group with anorexia nervosa (AN) in forced-bid trials with FDR-correction (q).

		EDI-3 ED	IUS
**Ventral anterior cingulum, right**	r	0.166	-0.215
	q	0.926	0.870
**Ventral anterior cingulum, left**	r	0.151	-0.299
	q	0.926	0.849
**Inferior orbito-frontal gyrus, right**	r	-0.094	-0.232
	q	0.926	0.870
**Inferior orbito-frontal gyrus, left**	r	0.122	-0.018
	q	0.926	0.942
**Medial orbitofrontal gyrus, right**	r	0.036	-0.374
	q	0.999	0.849
**Medial orbitofrontal gyrus, left**	r	0.128	-0.340
	q	0.926	0.849
**Middle orbito-frontal gyrus, right**	r	0.000	-0.253
	q	0.999	0.870
**Middle orbito-frontal gyrus, left**	r	0.009	-0.294
	q	0.999	0.849
**Caudate head, right**	r	0.407	-0.117
	q	0.822	0.893
**Caudate head, left**	r	0.376	-0.140
	q	0.822	0.893
**Nucleus accumbens, right**	r	0.231	-0.144
	q	0.926	0.893
**Nucleus accumbens, left**	r	0.485	0.090
	q	0.822	0.894

Abbreviations. EDI-3, Eating Disorder Inventory-3; ED, Emotion Dysregulation; IUS, Intolerance of Uncertainty.

Supplemental Table S3. Correlation between brain activation and behavior in the healthy control (HC) group in forced-bid trials with FDR-correction (q).

		EDI-3 ED	IUS
**Ventral anterior cingulum, right**	r	0.267	0.262
	q	0.511	0.816
**Ventral anterior cingulum, left**	r	0.164	0.331
	q	0.735	0.793
**Inferior orbito-frontal gyrus, right**	r	0.163	0.039
	q	0.735	0.842
**Inferior orbito-frontal gyrus, left**	r	0.407	0.161
	q	0.449	0.816
**Medial orbitofrontal gyrus, right**	r	0.268	0.160
	q	0.511	0.816
**Medial orbitofrontal gyrus, left**	r	0.360	0.254
	q	0.449	0.816
**Middle orbito-frontal gyrus, right**	r	0.342	0.177
	q	0.449	0.816
**Middle orbito-frontal gyrus, left**	r	0.286	0.166
	q	0.511	0.816
**Caudate head, right**	r	0.190	0.075
	q	0.735	0.842
**Caudate head, left**	r	0.132	-0.056
	q	0.824	0.842
**Nucleus accumbens, right**	r	0.061	-0.059
	q	0.871	0.842
**Nucleus accumbens, left**	r	0.044	0.322
	q	0.871	0.793

Abbreviations. EDI-3, Eating Disorder Inventory-3; ED, Emotion Dysregulation; IUS, Intolerance of Uncertainty.

Supplemental Table S4. Correlation between brain activation and behavior in free- minus forced-bid trials in the AN group with FDR-correction (q).

		EDI-3 ED	IUS
**Ventral anterior cingulum, right**	r	-0.320	-0.074
	q	0.420	0.926
**Ventral anterior cingulum, left**	r	-0.398	-0.050
	q	0.291	0.941
**Inferior orbito-frontal gyrus, right**	r	-0.100	0.019
	q	0.701	0.941
**Inferior orbito-frontal gyrus, left**	r	-0.190	-0.227
	q	0.493	0.701
**Medial orbitofrontal gyrus, right**	r	-0.217	0.074
	q	0.454	0.926
**Medial orbitofrontal gyrus, left**	r	-0.234	0.193
	q	0.438	0.723
**Middle orbito-frontal gyrus, right**	r	-0.252	-0.022
	q	0.438	0.941
**Middle orbito-frontal gyrus, left**	r	-0.473	-0.129
	q	0.165	0.845
**Caudate head, right**	r	-0.536	-0.354
	q	0.096	0.579
**Caudate head, left**	r	**-0.645**	-0.435
	q	**0.047**	0.579
**Nucleus accumbens, right**	r	-0.569	-0.131
	q	0.077	0.845
**Nucleus accumbens, left**	r	**-0.788**	-0.589
	q	**0.003**	0.181

Abbreviations. EDI-3, Eating Disorder Inventory-3; ED, Emotion Dysregulation; IUS, Intolerance of Uncertainty.

Supplemental Table S5. Correlation between brain activation and behavior in free- minus forced-bid trials in the healthy control (HC) group with FDR-correction (q).

		EDI-3 ED	IUS
**Ventral anterior cingulum, right**	r	0.062	-0.121
	q	0.800	0.910
**Ventral anterior cingulum, left**	r	0.109	-0.120
	q	0.800	0.910
**Inferior orbito-frontal gyrus, right**	r	0.342	0.097
	q	0.169	0.910
**Inferior orbito-frontal gyrus, left**	r	-0.071	-0.022
	q	0.800	0.910
**Medial orbitofrontal gyrus, right**	r	0.043	-0.235
	q	0.829	0.910
**Medial orbitofrontal gyrus, left**	r	0.072	-0.172
	q	0.800	0.910
**Middle orbito-frontal gyrus, right**	r	-0.096	-0.061
	q	0.800	0.910
**Middle orbito-frontal gyrus, left**	r	-0.123	-0.040
	q	0.797	0.910
**Caudate head, right**	r	0.354	-0.035
	q	0.169	0.910
**Caudate head, left**	r	0.275	0.033
	q	0.282	0.910
**Nucleus accumbens, right**	r	0.374	0.028
	q	0.169	0.910
**Nucleus accumbens, left**	r	0.227	-0.214
	q	0.403	0.910

Abbreviations. EDI-3, Eating Disorder Inventory-3; ED, Emotion Dysregulation; IUS, Intolerance of Uncertainty.

Supplemental Figure S1. Brain response to low and high bid across the study groups in anterior cingulate and nucleus accumbens.

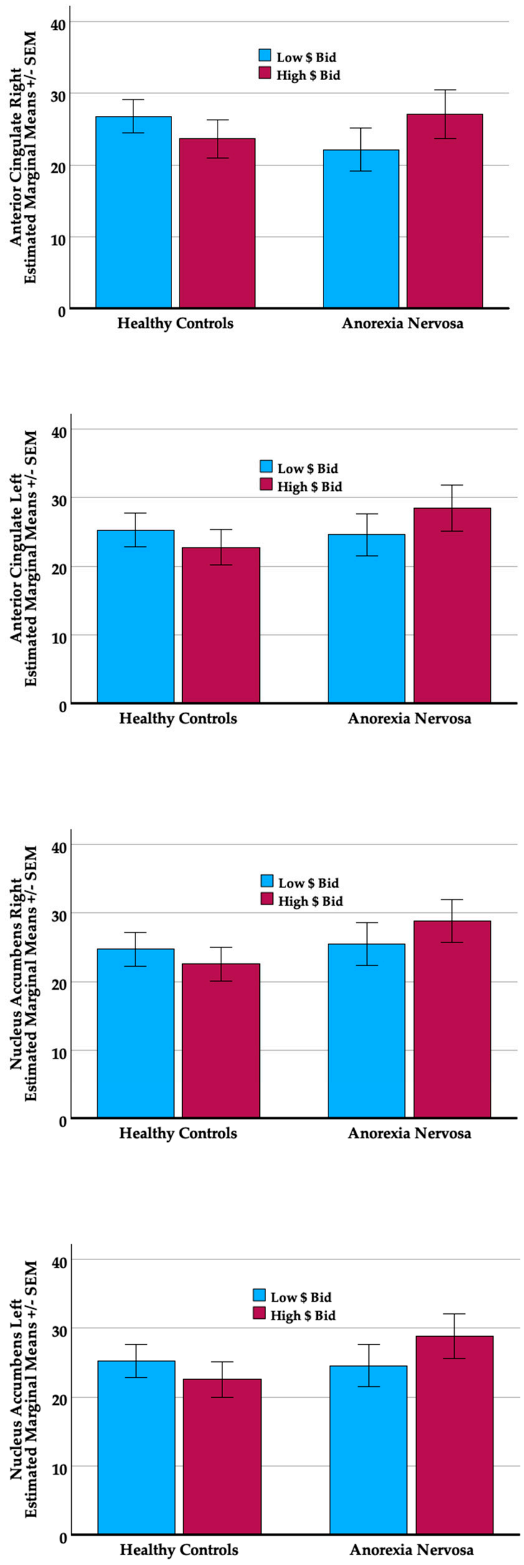

Supplement: Supplementary file 1 [file nutrients-16-03115-s001.zip › nutrients-3161863-supplementary.pdf]
